# Prognosis of Patients Over 60 Years Old With Early Rectal Cancer Undergoing Transanal Endoscopic Microsurgery – A Single-Center Experience

**DOI:** 10.3389/fonc.2022.888739

**Published:** 2022-06-14

**Authors:** Mingqing Zhang, Yongdan Zhang, Haoren Jing, Lizhong Zhao, Mingyue Xu, Hui Xu, Siwei Zhu, Xipeng Zhang

**Affiliations:** ^1^ Nankai University School of Medicine, Nankai University, Tianjin, China; ^2^ Department of Colorectal Surgery, Tianjin Union Medical Center, Tianjin, China; ^3^ Colorectal Cancer Screening Office, Tianjin Institute of Coloproctology, Tianjin, China; ^4^ The Institute of Translational Medicine, Tianjin Union Medical Center of Nankai University, Tianjin, China

**Keywords:** early rectal cancer, local recurrence, adjuvant therapy, transanal endoscopic microsurgery, 5-year overall survival

## Abstract

**Aim:**

Transanal endoscopic microsurgery (TEM) is widely performed in early rectal cancer. This technique offers greater organ preservation and decreases the risk of subsequent surgery. However, postoperative local recurrence and distant metastasis remain challenges for patients with high-risk pathological factors. This single-center study reports the prognosis of early rectal cancer patients over 60 years old after TEM.

**Methods:**

The data of the patients over 60 years old who underwent local anal resection were collected retrospectively. Moreover, the 5-year follow-up data were analyzed to determine the 5-year DFS and OS.

**Results:**

47 early rectal cancer patients over 60 years old underwent TEM. There were 27 patients with high-risk factors and 20 patients without high-risk factors. Two patients underwent radical surgery after TEM and ten patients received adjuvant treatment. Local recurrence occurred in 7 patients, of which 4 underwent salvage surgery. The 5-year progression-free survival rate was 75.6%, which was lower in the high-risk patients group (69.6%) than in the non-high-risk patients group (83.3%) (*P*>0.05). The 5-year OS was 90.2%, but there was no statistically significant difference between the two groups (high-risk patients 87.0%, non-high-risk patients 94.4%). Furthermore, there was no significant difference in DFS and OS between people over and under 70 years old.

**Conclusion:**

Some high-risk factor patients over 60 years old do not have inferior 5-year DFS and OS to the non-high-risk patients. TEM is an option for old patients with high surgical risks. Even if postoperative pathology revealed high-risk factors, timely surgical treatment after local recurrence would be beneficial to improve the 5-year DFS and OS.

## Introduction

Transanal local resection is one of the commonly employed surgical approaches for early rectal cancer. It is also recommended by many clinical guidelines for the treatment of early rectal cancer. Among these therapies, transanal endoscopic microsurgery (TEM) is the most commonly performed. Currently, TEM is mainly performed on patients with T1 low-risk rectal cancer (1). Generally speaking, the following criteria are favorable for TEM (2): lesions accounting for less than 30% of the rectal circumference; largest diameter of the tumor less than 3cm; distance between the margin and the tumor greater than 3mm; mobile tumor (not fixed); less than 8cm from the anal margin; T1 tumor; no vascular, lymphatic or nerve infiltration; highly to moderately differentiated. Other criteria where TEM can be considered include: no signs of lymph node metastasis in imaging examination; polyps resected under endoscopy showed cancerous infiltration, or the pathological examination results were uncertain; additional extended local resection was required; full-thickness resection was achieved.

With its excellent oncological and surgical safety (3), TEM allows for full-thickness resection and suture under direct vision (4, 5). Compared with radical surgery, TEM has been widely used for its superior anal function protection, low operation risk and fast postoperative recovery (6–9). The technique is especially popular with elderly patients and patients requesting anus preservation (10, 11). However, the risk of local recurrence and distant metastasis after TEM is still present (4). Clinical studies indicated that the local recurrence rate of T1stage rectal cancer after TEM was about 10% (12), and the 5-year local recurrence rate of the patients with high risk or incomplete resection exceeded 30% (13). The patients with pathological high-risk factors were generally recommended to pursue additional radical surgery after TEM (14). For those high-risk patients who failed to undergo additional radical surgery, the prognosis after TEM deserved further evaluation (15). This study aimed to investigate the prognosis of the patients over 60 years old who underwent TEM in our center, regardless of the presence or absence of postoperative pathological high-risk factors.

## Method

A retrospective analysis was conducted based on the collected data of the early rectal cancer patients over 60 years old who underwent TEM at the department of colorectal surgery of Tianjin Union Medical Center from January 2011 to January 2016. Patients with a histopathological diagnosis of malignancy were enrolled. Cases of severe dysplasia and carcinoma *in situ* and neoadjuvant chemoradiotherapy were excluded. All patients underwent preoperative digital rectal examination and colonoscopy. The local tumor stage and lymph node status were assessed by pelvic MRI or transrectal ultrasound, and distant metastasis was assessed by chest and abdominal CT scan. There was no evidence of lymph node involvement and distant metastasis in preoperative imaging.

The operation was performed by three surgeons with extensive experience in TEM surgery. All procedures were performed under general anesthesia, as previously described (16, 17). Resection margins were marked to incorporate a 1-cm cuff of normal mucosa around the location where cancer was known or suspected. In most patients, a full-thickness resection was performed. Closure of the rectal wall defect was routinely performed.

All patients were diagnosed with early rectal cancer (pT1-T2) according to the *UICC/AJCC T Staging System (Seventh Editio*n) for pathological staging. pT1 tumors were evaluated according to the Kikuchi submucosal staging system (Sm1-3). The resection margin was considered positive when the cancer was located within 1 mm of the specimen’s resection margin.

The collected data included demographic information, preoperative staging, intrarectal ultrasonography, MRI, adjuvant therapy, surgical details, complications, tumor histopathology, postoperative management (additional radical surgery, radiotherapy or monitoring), recurrence and metastasis. The present study was approved by the Ethics Committee of Tianjin Union Medical Center.

### Case Follow-Up

Periodical re-examination was recommended in accordance with the clinical guidelines: The patients should be tested once every 3-6 months in the first 2 years, and once every 6 months in the 3rd to 5th years with a digital-anal examination, proctoscopy, transrectal ultrasound or MRI and CEA. Colonoscopy was performed within 1 year after surgery. Patients with progression-free survival of more than 5 years were followed up by telephone. All patients completed the LARS scoring questionnaire (18) to evaluate postoperative anal defecation function.

The main outcome indicator was the 5-year overall survival (OS). The secondary outcome indicators included the 5-year disease-free survival time (DFS) and the 5-year recurrence and metastasis rate. OS and DFS were both measured from the initial TEM date. The observation endpoint of OS was the date of death or the time of the last follow-up; the observation endpoint of DFS was the date of the first diagnosis of recurrence or metastasis after the operation or the time of the last follow-up. The follow-up period ended on August 27, 2021.

### Statistical Analysis

Statistical analysis was performed using SPSS 23.0. The count data were represented by the number of cases (composition ratio), while the measurement data conformed to a normal distribution and were described by (x̄±s). Continuous variables not suiting a normal distribution were described as median (interquartile range [IQR]) and compared between groups using the non-parametric Wilcoxon rank-sum test. The Kaplan-Meier method was employed to draw the survival curve of the patients, and the Log-rank test was adopted to compare the survival curves. *P*<0.05 was considered statistically significant throughout this study.

## Research Results

**Figure 1:**
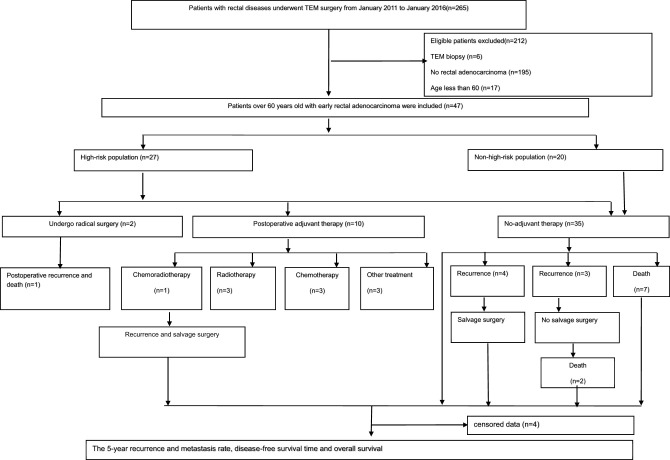
Research schematic of the patients with early rectal cancer undergoing TEM surgery.

During the 5-year period from January 2011 to January 2016, 265 TEM procedures were recorded. 47 early rectal cancer patients over 60 years old underwent TEM, including 20 males and 27 females, with an average age of 69.9 years (ranging from 60 to 88 years old). Adenocarcinoma was confirmed by preoperative pathological biopsy in 25 patients. The mean distance between the lesion and the anal verge was 5.4cm (range 4-12cm), and the preoperative staging evaluated by transrectal ultrasound/MRI was no later than cT1N0M0 in all patients. The detailed screening workflow is shown in **Figure 1**, and the demographic data and tumor characteristics are displayed in **Table 1**.

**Table 1:** Demographic and tumor characteristics of 47 patients with rectal cancer who underwent TEM.

	Patient number
All patients, n (%)	47 (100%)
Sex, n (%)	
Male	20 (42.6%)
Female	27 (57.4%)
Age	
<70, years	25 (53.2%)
≥70, years	22 (46.8%)
Median age, years	69.0
Mean age, years	69.9
Age range, years	60-88
Tumor diameter, n (%),cm	
<=1.0	3 (6.4%)
1.1-2.0	18 (38.3%)
2.1-3.0	11 (23.4%)
3.1-4.0	6 (12.8%)
4.1-5.0	4 (8.5%)
>5.0	1 (2.1%)
N/A^b^	4 (8.5%)
Median tumor diameter, cm	2.5
Primary tumor (T) category	
T1	37 (78.7%)
T2	10 (21.3%)
Differentiation	
Well-differentiated	2 (4.3%)
Moderately Well-differentiated	3 (6.4%)
Moderately differentiated	41 (87.2%)
Moderately Poorly-differentiated	1 (2.1%)
Pathological pattern	
Adenocarcinoma	41 (87.2%)
Myxoadenocarcinoma and signet ring cell and papillary carcinoma	6 (12.8%)
Vascular invasion (V)	
V0	44 (93.6%)
V1	3 (6.4%)
Resection margin	
positive	4 (8.5%)
negative	43 (91.5%)
Sm3 (T1)	
positive	15 (31.9%)
negative	22 (46.8%)
Post-TEM management	
Treatment before recurrence and metastasis	
YES	10 (21.3%)
NO	36 (76.6%)
N/A	1 (2.1)
Observation	
YES	45 (95.7%)
NO	2 (4.3%)

(Some data items are incomplete, and the percentage reflects the number of objects available for the data item.)

Characteristics of the CRC patient cohort (47 patients). Treatment before recurrence and metastasis of CRC patients consisted of adjuvant radio-chemotherapy in 2.1% (1/47), chemotherapy in 6.4% (3/47), adjuvant radiotherapy in 6.4% (3/47), Other treatment in 6.4% (3/47), No treatment in 78.7% (37/47). N/A, data not available.

### Pathology

The postoperative pathological staging was as follows: 37 cases (78.7%) were T1 and 10 cases (21.3%) were T2. There were 41 cases (87.2%) of adenocarcinoma, 4 cases (8.6%) of mucinous adenocarcinoma, 1 case (2.1%) of signet ring cell carcinoma, and 1 case of micropapillary carcinoma (2.1%). Considering of pathological risk factors, only one case (2.1%) demonstrated moderate-poor differentiation. A vascular tumor thrombus was found in 3 (6.4%) cases, and positive tumor margins were found in 4 (8.5%) cases. In addition, retrospective analysis of the pathological sections showed that 15 patients (31.9%) with stage T1 were Sm3. There were 27 patients (collectively called high-risk patients, 57.4%) with pathological risk factors (poor differentiation, positive resection margin, T2 stage and above, vascular invasion, Sm3 etc.) and 20 non-high-risk patients (42.6%). There was no significant difference in age and gender between the two groups (**Table 2**).

**Table 2:** Comparison of the baseline characteristics between high-risk and non-high-risk patients.

Variables		Early rectal cancer		χ^2^ value/Z	*P-value*
		High-risk, n (%)	Non high-risk, n (%)		
Age	<70	16 (59.3)	9 (45.0)	0.938	0.333
	≥70	11 (40.7)	11 (55.0)
Gender	Male	12 (44.4)	8 (40.0)	0.093	0.761
	Female	15 (55.6)	12 (60.0)
Follow-up time		101.0 (32.0)	76.5 (30.3)	-1.829	0.407

### Subsequent Treatment for High-Risk Patients

According to the postoperative pathological results, pT1N0 patients with pathological risk factors and pT2 patients were recommended to undergo radical rectal cancer surgery or conventional fractional concurrent chemoradiotherapy (radiation therapy of 50-54Gy for 25-30 times and/or capecitabine or other chemotherapy). Among them, 2 patients agreed to undergo radical surgery and 10 patients received adjuvant therapy.

#### Radical Surgery

Two patients with pathological factors (both T2 stage) underwent early radical surgery in the first week following TEM as per the doctor’s recommendation. 1 case of low anterior rectal resection had anastomotic recurrence 4 months after radical surgery and died of other causes 28 months after TEM. The other case (rectal abdominoperineal resection) had no disease recurrence within the 111 months of follow-up after TEM. These two patients underwent radical surgery within a short period of time after TEM, which is not reflective of TEM surgery efficacy. Thus, they were excluded from the subsequent analysis of local recurrence, metastasis and survival.

#### Adjuvant Therapy

Ten patients received adjuvant treatment after TEM, in which 1 case received radiotherapy combined with chemotherapy (2.1%), 3 cases received chemotherapy (6.4%), 3 cases received radiotherapy (6.4%), and 3 cases received other treatment (traditional Chinese medicine, etc., 6.4%). The patient receiving combined radiotherapy and chemotherapy developed local recurrence and a secondary malignant tumor in the perineum 32 months after TEM surgery. Rectal abdominoperineal resection was then performed, and the patient was still alive at the end of the follow-up period. Among the 3 patients receiving chemotherapy, 2 patients survived, while the other patient died 55 months after TEM. Furthermore, 2 of the 3 patients who received radiotherapy were alive, whereas 1 case died 61 months after TEM. Among the 3 patients who received other treatments, 2 were alive; 1 case had a local recurrence 4 months after TEM, underwent low anterior rectal resection, and died 28 months after TEM.

### Follow Up: Local Recurrence and Distant Metastasis

**Figure 2:**
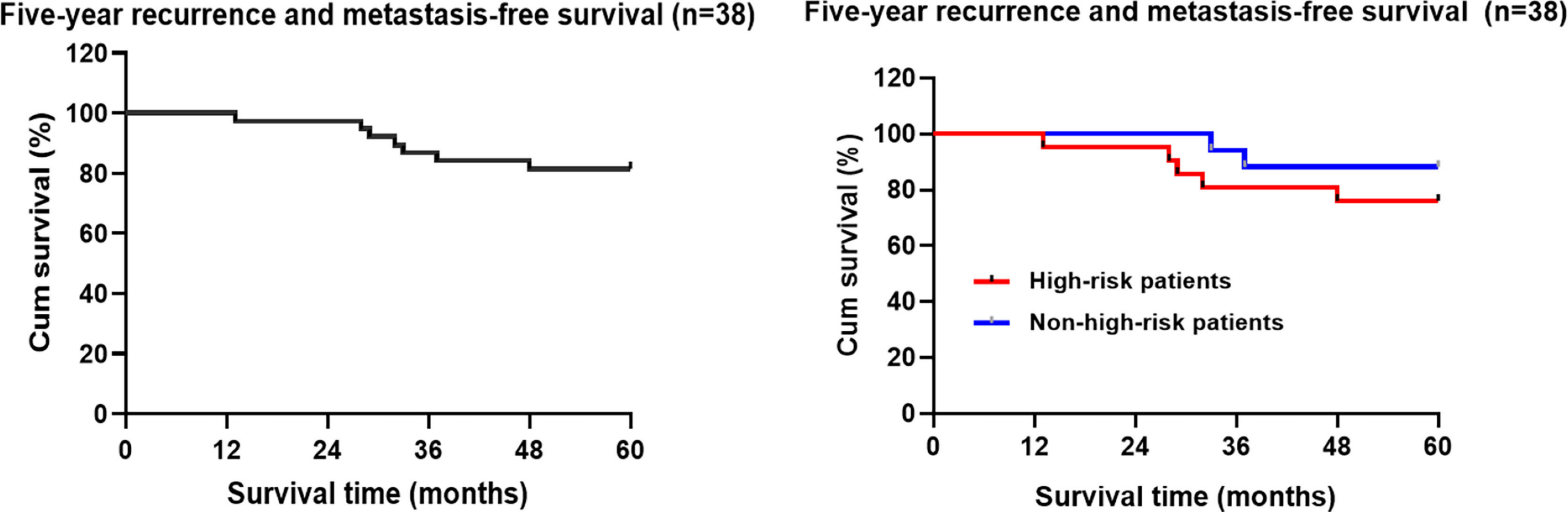
Kaplan-Meier analysis of the follow-up five-year recurrence and metastasis free-survival rate of the patients with early rectal cancer undergoing TEM.

Among the 45 patients who underwent TEM, a total of 7 (15.6%) patients had recurrence and metastasis at the end of the follow-up period, including 6 cases of local recurrence (2 cases with lung metastasis, 1 case with perineal metastasis) and 1 case with isolated liver metastasis. The median time of recurrence and metastasis after TEM was 32 months (ranging from 13 to 48 months). According to the Kaplan-Meier statistical analysis, the 5-year recurrence and metastasis rate was 18.4% (7/38) (95% CI: 50.972-58.502). The 5-year recurrence rate of T2 patients was 28.6% higher than that of T1 patients at 16.1%, but the difference was not statistically significant (χ^2 ^= 0.657, *P*=0.418). A higher 5-year recurrence and metastasis rate was observed in the high-risk population (23.8%) than in the non-high-risk population (11.8%), but the difference was not statistically significant (χ^2^ = 0.997, *P* = 0.318) (**Figure 2**).

### Surgical Treatment After Recurrence and Metastasis

Among the 7 patients with recurrence and metastasis, 4 cases underwent rectal abdominoperineal resection after local rectal recurrence. The median time of operation was 36.0 months (ranging from 32.0 to 48.0 months), and the pathology after additional surgery revealed a negative surgical margin. After the recurrence, 1 patient received radiotherapy and chemotherapy before the surgery. One year after the surgery, a secondary malignant tumor was found in the lung, and chemotherapy with mFOLFOX6 was given. Besides, 1 patient with recurrence was not given treatment after the surgery, but no new recurrence or metastasis occurred. 1 patient underwent abdominoperineal resection for rectal cancer after the recurrence, a secondary malignant tumor appeared in the perineum 8 months after the operation, and surgical resection was performed again. 1 patient underwent a second TEM after the recurrence, and abdominoperineal resection for rectal cancer was performed due to local recurrence 3 months after the surgery, and postoperative chemotherapy was given. The other 3 patients with recurrence and metastasis did not undergo further surgery.

### Follow-Up Instructions

During the 5-year follow-up, pathological features of adenocarcinoma were identified in 4 pT1 patients, with moderately differentiated, non-lymphovascular invasion, and negative margin; two of the patients were Sm3 and two of them received postoperative radiotherapy. None of the patients had abnormal anal function.

### Disease-Free Survival Time

**Figure 3:**
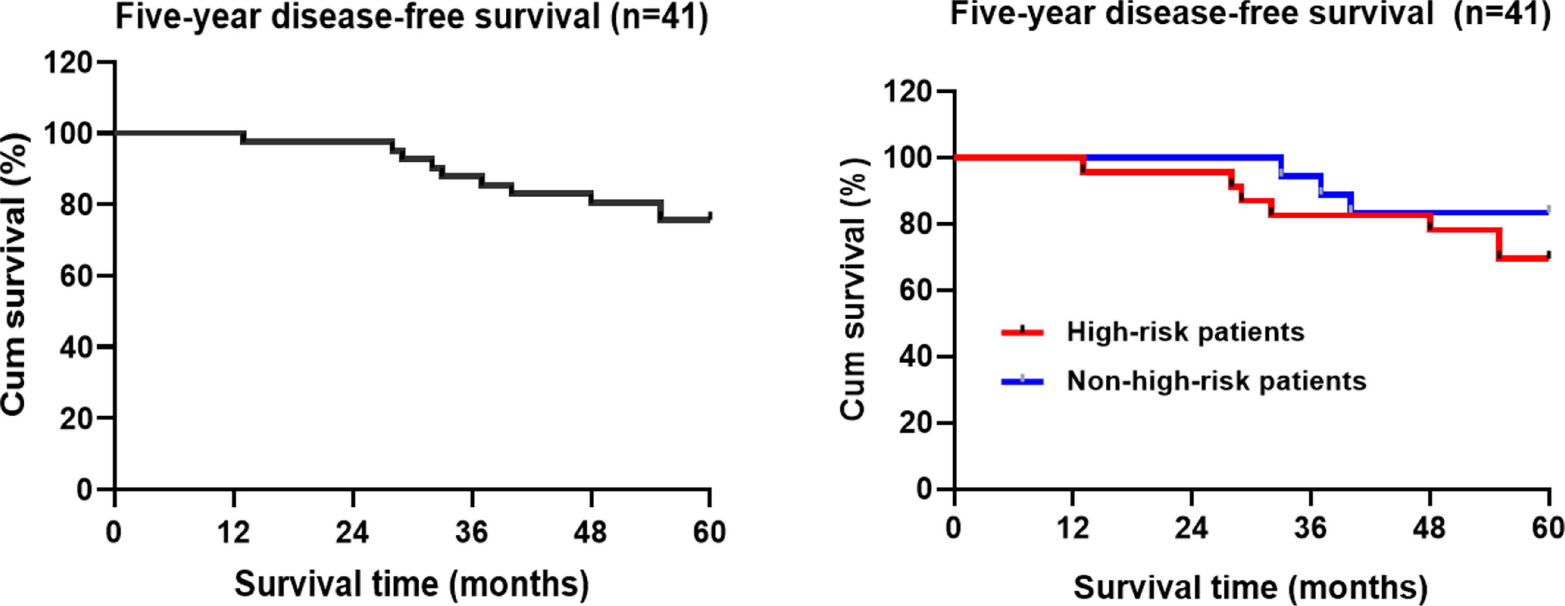
Kaplan-Meier analysis of the five-year disease-free survival rate of TEM patients with early rectal cancer.

Kaplan-Meier statistical analysis indicated that the 5-year progression-free survival rate of the patients was 75.6% (31/41) (95%CI: 50.832-57.94) (**Figure 3**). Further analysis of the clinicopathological characteristics of the patients revealed differences in gender, age, postoperative adjuvant treatment, pathological T staging, pathological type, and Sm3, but the differences were not statistically significant. This may be related to the small sample size of this group. In contrast, there were statistically significant differences in tumor diameter. A lower 5-year DFS was observed in high-risk patients (69.6%) compared to non-high-risk patients (83.3%) (*P*>0.05) (**Table 3** and **Figure 3**).

**Table 3:** Analysis of clinically related factors affecting the disease-free survival time of the patients with early rectal cancer.

Factors	Number of cases	Median disease-free survival time (range, month)	Disease-free survival rate		
			Five-year survival rate (%)	χ^2^ value	*P*-value
Gender				0.156	0.692
Male	15	84 (13~110)	73.3		
Female	26	91 (29~127)	76.9		
Age				0.098	0.754
≥70	19	75 (13~116)	73.7		
<70	22	87 (29~127)	77.3		
Postoperative adjuvant therapy				0.044	0.834
With	7	73 (32~107)	71.4		
Without	34	87 (13~127)	76.5		
Tumor diameter, cm				8.675	0.003
≥2.5	11	48 (13~127)	45.5		
<2.5	30	92 (28~116)	86.7		
Adenocarcinoma				1.491	0.222
Common type	37	87 (13~127)	78.4		
Special type	4	70 (29~114)	50.0		
Pathological stage				0.933	0.334
T1	33	85 (13~127)	78.8		
T2	8	74 (29~104)	62.5		
Vascular tumor thrombus				3.609	0.057
With	3	48 (32~110)	33.3		
Without	38	86 (12~127)	78.9		
Basement				1.719	0.190
Positive	4	75 (13~104)	50.0		
Negative	37	85 (28~127)	78.4		
Sm3 (T1)				0.452	0.502
positive	13	104 (28~114)	84.6		
negative	20	74 (13~127)	75.0		
Classifying Population according to risk factors				1.026	0.311
High-risk population	23	89 (13~114)	69.6		
Non-high-risk population	18	74.5 (33-127)	83.3		

### Overall Survival

**Figure 4:**
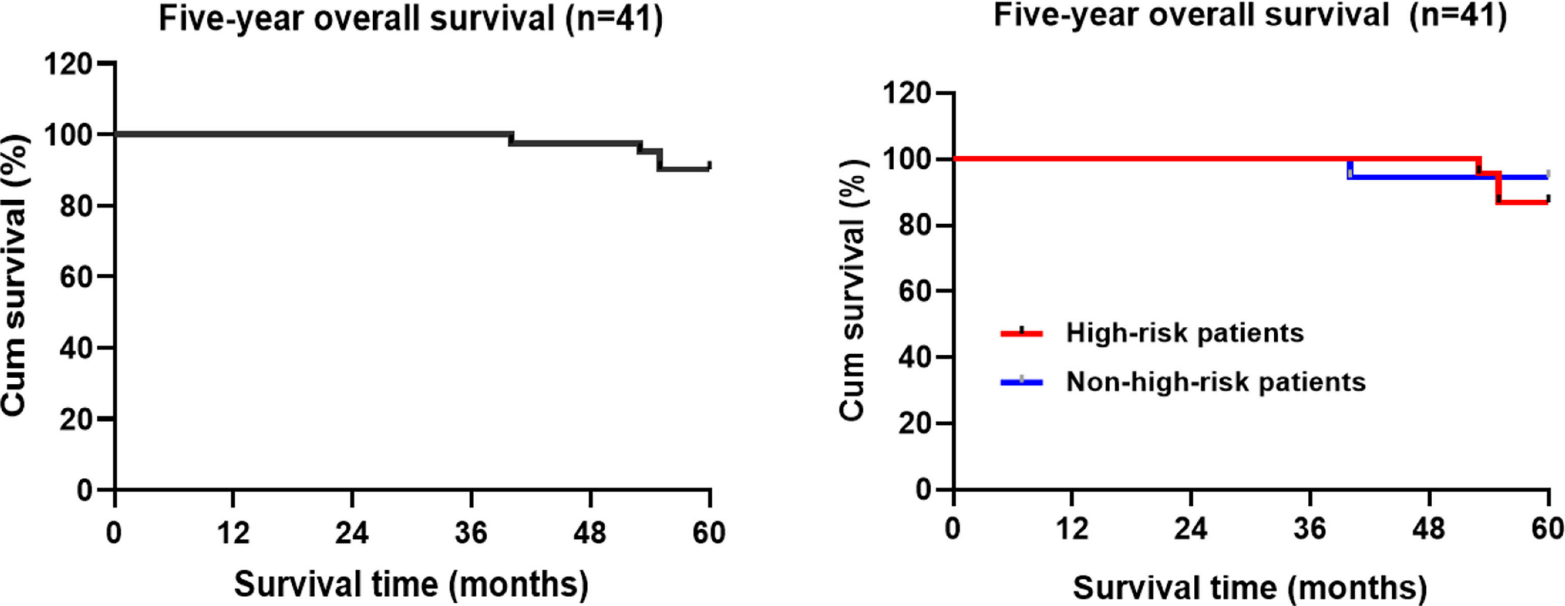
Kaplan-Meier estimated the overall survival rate of the patients with rectal cancer during the 5-year follow-up period.

The 5-year overall survival rate of the patients in this group was 90.9% (4/41) (95%CI:58.066-60.129) (**Figure 4**). Kaplan-Meier statistical analysis of the clinicopathological characteristics of the patients demonstrated no significant difference in gender, age, postoperative adjuvant treatment, tumor diameter, T stage, pathological type, presence of the tumor thrombus, Sm3, and positive basal lamina. A lower 5-year OS was observed in the high-risk population (87.0%) than in the non-high-risk population (94.4%), but the difference was not statistically significant (**Table 4** and **Figure 4**).

**Table 4:** Analysis of clinically related factors affecting the overall survival time of the patients with early rectal cancer.

Factors	Number of cases	Median Overall survival time (range, month)	5-year overall survival rate (%)	χ^2^ value	*P*-value
Gender				0.427	0.513
Male	15	85 (40~110)	86.7		
Female	26	99.5 (55~127)	92.3		
Age				1.514	0.219
≥70	19	78 (40~116)	84.2		
<70	22	101 (55~127)	95.5		
Postoperative adjuvant therapy				0.152	0.696
With	7	85 (55~123)	85.7		
Without	34	88.5 (40~127)	91.2		
Tumor diameter, cm				0.001	0.971
≥2.5	11	87 (40~127)	90.9		
<2.5	30	92 (53~116)	90.0		
Adenocarcinoma				0.999	0.318
Common type	37	92 (40~127)	91.9		
Special type	4	75 (55~114)	75.0		
Pathological stage				0.060	0.807
T1	33	88 (40~127)	90.9		
T2	8	94 (55~123)	87.5		
Vascular tumor thrombus				0.331	0.565
With	3	110 (101~123)	100.0		
Without	38	87 (40~127)	89.5		
Basement				0.999	0.318
Positive	4	81(55~104)	75.0		
Negative	37	88 (40~127)	91.9		
Sm3 (T1)				0.921	0.337
positive	13	104 (53~114)	84.6		
negative	20	76.5 (40~127)	95.0		
Classifying Population according to risk factors				0.558	0.455
High-risk population	23	101 (53~123)	87.0		
Non-high-risk population	18	76.5 (40-127)	94.4		

## Discussion

T1 rectal cancers without pathological risk factors may be effectively treated with TEM without jeopardizing long-term oncologic outcomes (19). In contrast, radical surgery is recommended in high-risk pathological stage T1 (pT1) or pT2 rectal cancer. However, older patients with significant comorbidity may not be viable candidates for radical surgery (20). The patients in this group were generally older (the average age was 69.9 years) and some elderly patients could not tolerate radical surgery or refused to undergo combined abdominal and perineal radical surgery, even in the presence of pathological risk factors. Therefore, some patients with larger tumors (the maximum diameter was 6.0cm) opted for TEM. Through follow-up, it was found that the 5-year OS of the patients reached 90.2%, with a favorable overall prognosis, demonstrating that TEM is a worthwhile option for elderly patients with early rectal cancer.

Significant differences in the recurrence rate between T1 and T2 patients after TEM surgery have been reported. The 5-year local recurrence rate of T2 patients was as high as 29.3 ~ 47% (21), and the lymph node involvement rate of T2 tumors was also about twice higher than that of the T1 stage (22, 23). Therefore, T2 patients should receive more active surgical treatment (24). This was also consistent with our findings. Although the results were not statistically significant, the 5-year recurrence and metastasis rate in T2 patients (28.6%) higher than that in T1 patients (16.1%) (25). Meanwhile, T1 rectal cancer also carries the risk of lymph node metastasis (26, 27). Signs of lymph node metastasis before surgery would also directly affect the treatment program (19, 28, 29). In the present study, all patients underwent preoperative MRI or intrarectal ultrasound, chest and abdominal CT scan, and no evidence of lymph node involvement and distant metastasis was found.

A detailed pathological examination should be conducted for the tissue specimens resected by TEM. For the patients with poor differentiation, large tumors, vascular invasion and other risk factors (30), an increased local recurrence rate was observed, ranging from 20% to 30% (31). Therefore, if the postoperative pathological examination showed poor histological differentiation, positive margins, lymphatic and vascular invasion, T2 and above stages, etc., additional radical surgery is recommended (32). For patients with the above-mentioned high-risk factors who refused to undergo secondary surgical resection and had unclear lymph node status, the subsequent treatment plan should be determined after multidisciplinary discussion (33).

Complete tumor resection greatly reduces the risk of local recurrence (13). Previous studies indicated that the low risk of local recurrence of rectal cancer after TEM was mostly caused by the tumor residues from the previous resection rather than from undetermined lymph node metastasis (34). Therefore, full-thickness resection, adequate resection margins and en-bloc resection are performed to achieve adequate oncological safety (35). When the tumor cannot be completely removed, such as positive margins, additional radical surgery is currently recommended (36). Studies have shown no difference in outcomes with primary TME surgery (37). Considering the relationship between the depth of invasion and lymph node metastasis and local recurrence, most scholars recommend radical surgery for patients with stage T1 rectal cancer with deep submucosal invasion (Sm3 or > 1 mm) (30), despite other studies reporting Sm3 was not related to lymph node metastasis and survival (38). In this study, Sm3 was not included in the treatment guidelines at the time of treatment for some patients. We performed a retrospective analysis and found that the DFS and OS of Sm3 patients in T1 patients were similar to those of other T1 patients. However, this finding may be related to the small sample size.

Currently, the optimal treatment measures for patients with pathological high-risk factors are controversial (15, 24, 39). Most experts still recommend additional radical surgery, but other treatment measures are also worth exploring, such as adjuvant radiotherapy (40, 41)or extended local resection for patients with a high risk of recurrence (42). However, the previous studies pointed out that even if the patients were treated with total mesorectal excision (TME), the efficacy of the radical surgery would be lower than that of direct TME due to the effects of the initial TEM (43). Nevertheless, it is not associated with increased morbidity or mortality. Immediate laparoscopic TME after TEM for rectal cancer may result in a significantly increased risk of APR (44). Furthermore, the fibrotic scar caused by full-thickness TEM hinders the maintenance of proper surgical planes during TME surgery (45). Therefore, some researchers suggest that TME surgery following TEM excision is best delayed for 6 to 12 weeks in order to reduce postsurgical inflammation (29).

In this data group, the 5-year OS of the patients with recurrence after TEM and additional radical surgery were not inferior to those of low-risk patients without recurrence. The results indicated that the additional radical surgery after the recurrence with TEM surgery could improve the prognosis of the patients. This is consistent with some other observations and conclusions (37, 46, 47). Consequently, TEM can be used in exceptional cases with high-risk factors when the patient is not fit for radical surgery (45).

In one study including 33 patients who received adjuvant radiotherapy due to poor histopathological characteristics with a median follow-up of 3.2 years, the results indicated that 3 patients (9.1%) had local recurrence, and the estimated 1-year and 3-year local recurrence rates were 0% and 6.9%, respectively (40). The above protocol was also offered to the patients in the present study.

In another study, additional TEM was performed on part of the patients. It was found that the safety and radical effect of the additional TEM was nearly the same as that of the first operation (48, 49). In this study, four patients had positive margins; all of the tumors were greater than 2 cm in diameter (2.5-4 cm) and were close to the anus, increasing the risk of impaired anal function for additional surgery. The patients refused the recommended repeat TEM surgery or radical surgery and received postoperative adjuvant therapy. Although the anorectal function after repeated TEM is preserved (50), many factors including malignant lesions (51, 52), excessive circumferential mucosal defects (53), and closer proximity of the tumor to the anal verge suggest a higher risk of impaired anal function after TEM.

In addition, this study found no statistically significant differences in recurrent metastases and overall survival with or without adjuvant therapy after TEM. These findings are similar to the results of a recent study, concluding that survival after transanal local excision with or without chemoradiotherapy is comparable to that of TME, while TEM allows better bowel function and postoperative quality of life (54).

The follow-up program should be determined according to the risk of tumor recurrence and metastasis. Clinical studies indicated that the recurrence in T1 patients with local resection mostly occurred within 1 to 2 years after the surgery. Recurrence was usually located at the site of the primary lesions, and more than one-third of the patients recurred at the site outside the intestinal cavity (55). Therefore, most studies limit the duration of close follow-up to the initial 1 to 2 years (35, 56). It is generally believed that the patients should be reviewed every 3 months in the first 2 years (57). The examination included digital rectal examination, rigid sigmoidoscopy or proctoscope, ERUS and pelvic MRI, and monitoring carcinoembryonic antigen levels (58). Some scholars suggested that a CT examination of the chest and abdomen should be performed every 6 months to exclude distant metastasis (59). The postoperative follow-up of patients was performed according to guideline recommendations. Most of the patients who developed local recurrences had no obvious clinical symptoms. As a consequence, it is essential to optimize the follow-up program and closely monitor for local recurrence. The routine application of ERUS and pelvic MRI should be recommended (60).

This study has several main limitations, including the retrospective design and small sample size. Furthermore, due to various factors, some patients did not comply with the recommendations to receive postoperative adjuvant therapy, which may affect the conclusion of the study.

TEM surgery provides prominent advantages in the treatment of early rectal cancer, preserving anal function and imposing lower surgical risk and surgical pain. The results of the present study suggest a favorable overall prognosis for early rectal cancer patients over 60 years old who underwent TEM. For patients with postoperative pathological high-risk factors, additional radical surgery after local recurrence could also effectively improve the prognosis of the patients. Moreover, the stratified analysis demonstrated no difference in DFS and OS in patients above 70 years. TEM surgery may be an option for older patients with high surgical risk. With the promotion of neoadjuvant therapy, the application of TEM in patients with T2 or T3 stage tumors is being explored (21, 61–63). Further research on this topic will enable a broader application of TEM, which will play an essential role in the treatment of rectal cancer.

## Conclusion

TEM is widely performed in early rectal cancer and also can be used in patients with high-risk factors who are not fit for radical surgery. Elderly patients tend to opt for TEM as it allows organ preservation and is a relatively safe surgery. However, these data remain to be confirmed. We report the prognosis of early rectal cancer patients over 60 years old. As an alternative for elderly rectal cancer patients with or without pathological high-risk factors, TEM is a reliable and effective therapeutic option.

## Data Availability Statement

The original contributions presented in this study are included in the article. Further inquiries can be directed to the corresponding author.

## Ethics Statement

The studies involving human participants were reviewed and approved by the Ethics Committee of Tianjin Union Medical Center. Written informed consent for participation was not required for this study in accordance with the national legislation and the institutional requirements.

## Author Contributions

SZ, XZ and MZ conceived the idea for the study. XZ and MZ were involved in planning and supervised data collection. HJ, YZ and LZ performed data collection. MZ, YZ and LZ conducted data analysis. MZ, YZ drafted the manuscript. MX and HX contributed to writing of manuscript. All authors have discussed and decided that this manuscript is the final version and agreed to publish it.

## Funding

This study was funded by Foundation of Tianjin Union Medical Center (grant number: 2016YJZD002 and 2016RMNK002). This work was funded by Tianjin Key Medical Discipline (Specialty) Construction Project.

## Conflict of Interest

The authors declare that the research was conducted in the absence of any commercial or financial relationships that could be construed as a potential conflict of interest.

## Publisher’s Note

All claims expressed in this article are solely those of the authors and do not necessarily represent those of their affiliated organizations, or those of the publisher, the editors and the reviewers. Any product that may be evaluated in this article, or claim that may be made by its manufacturer, is not guaranteed or endorsed by the publisher.
